# Assessing effects of low‐dose CT attenuation correction on quantification and image quality on a LAFOV PET system with a modified NEMA‐IQ phantom

**DOI:** 10.1002/mp.70706

**Published:** 2026-11-01

**Authors:** Vanessa Nadig, Jane Mackewn, Georgios Krokos, Lewis C. Davies, Daniel Darian, Sarah‐May Gould, Gary J. R. Cook, Alexander Hammers, Andrew J. Reader, Paul K. Marsden

**Affiliations:** ^1^ Siemens Healthcare Limited Camberley UK; ^2^ King's College London and Guy's & St Thomas' PET Centre School of Biomedical Engineering and Imaging Sciences King's College London London UK; ^3^ Department of Biomedical Computing School of Biomedical Engineering and Imaging Sciences King's College London London UK; ^4^ Department of Early Life Imaging, School of Biomedical Engineering and Imaging Sciences King's College London London UK; ^5^ Department of Imaging Physics and Engineering School of Biomedical Engineering and Imaging Sciences King's College London London UK

**Keywords:** LAFOV, low‐dose imaging, PET/CT, quantification, tin filter, total‐body PET

## Abstract

**Background:**

Over the past decades, efforts have been directed toward increasing the sensitivity of PET systems to enable low‐dose PET imaging and shorter scan times. The Biograph Vision Quadra PET/CT scanner (Siemens Healthineers, Knoxville, TN, USA) combines an increased solid angle coverage with time of flight, enabling a significant reduction in administered activity. To fully benefit from low‐dose PET imaging, the dose of the CT scan should ideally also be reduced.

**Purpose:**

The CT radiation dose can be reduced by lowering the tube voltage and exposure as well as by the use of tin filtration. Reducing the CT dose must be carefully aligned with any impact that changing CT image quality and quantitative metrics may have on the PET quantification, for example via linear attenuation coefficients. We evaluate the impact of low‐dose CT protocols on the PET quantification in soft‐tissue‐like and bone‐equivalent materials using a customized phantom.

**Methods:**

The phantom was assembled out of a NEMA‐IQ phantom, several layers of saline and two bovine long bones, to simulate a patient positioned with arms down. A 10‐min PET scan with 47 different accompanying CT scans was acquired. The CT tube voltage and exposure were varied between 70kVto140kV, with and without tin filtration, and 7mAsto400mAs, respectively. The effect of the different CT parameters on the PET image quality and quantitative accuracy was assessed via several quantitative metrics, including standardized uptake value (SUV) and signal‐to‐noise ratio, along with the CT dose length product (DLP).

**Results:**

The recorded DLP values suggest that using all tube voltages except 140kV combined with exposures in the range of 7mAsto30mAs, can result in dose savings of ≥ 80% compared to a typical clinical protocol (120kV, 50mAs). Compared to this typical protocol, HU differed more in bone (10%to40% difference) than in soft tissue (1%to30% difference), and more streak artefacts and photon starvation were seen when configuring low tube voltages and exposures, with an accompanying increase in CT noise. The observed artefacts were transferred with low intensity to the PET images via the attenuation map. The ${\mathrm{SUV}}_{\mathrm{mean}}$ remained within 2% of the ${\mathrm{SUV}}_{\mathrm{mean}}$ of the typical clinical protocol for most tested protocols. The presence of artefacts in the CT images had a bigger impact on PET quantification in both soft tissue and bone (${\mathrm{SUV}}_{\mathrm{mean}}$ difference of 10%to20%) than any difference in HU. Protocols that resulted in artefacts showed an increased inaccuracy in attenuation correction and scatter estimation, as assessed via the “lung” residual error (up to 10%to20% increase).

**Conclusions:**

Even for low‐dose CT protocols, PET quantification remained consistent in all NEMA‐IQ spheres for many materials. Beam hardening and photon starvation in the CT image are likely to occur in specific body regions such as hips, shoulders or the abdomen for an arms‐down scan and these effects had a bigger impact on the quantification than any difference in HU in both soft tissue and bone. This therefore should be considered when selecting suitable CT imaging parameters.

## 1 INTRODUCTION

Over the past decades, efforts have been dedicated to increase the sensitivity of PET systems to enable low‐dose PET imaging and shorter scan times. A higher sensitivity can be achieved by increasing the solid angle coverage of a PET system, either by extending the axial field of view (aFOV) of a scanner^1^, ^2^, ^3^ or building organ‐dedicated systems with the detectors closely aligned to the organ of interest.^4^, ^5^, ^6^ While the idea of an extended aFOV was first suggested in the 1990s,^7^, ^8^ the world's first total‐body PET scanner was built only recently in 2018,^1^, ^9^ followed by the Biograph Vision Quadra, a large‐axial‐field‐of‐view (LAFOV) PET released in 2020.^10^ The aFOV of current LAFOV PET systems ranges from 106cmto194cm,^1^, ^10^ covering the patient at least from apex to thighs or entirely for the larger scanners. The extended aFOV is expected to increase sensitivity for whole‐body imaging by up to 40‐fold in a 2‐m‐long scanner compared to a 20‐cm‐long scanner.^9^

The Biograph Vision Quadra PET/CT scanner (Siemens Healthineers, Knoxville, TN, USA) combines both an extended aFOV of 106 cm^10^ and a TOF resolution of 228 ps (FWHM),^10^ which has enabled substantial reductions in administered activity and scan time.^11^, ^12^ The radiation dose of the accompanying CT scan, typically in the order of about 7.5mSv, can constitute up to 50% of a typical PET/CT scan's overall dose,^13^ and becomes even more critical for LAFOV and total‐body PET scanners as they can often require the acquisition of a full‐body CT. To benefit from short and low‐dose PET scans and make them accessible to vulnerable groups, such as paediatric or pregnant patients, and unlock applications such as multi‐time point imaging, imaging of healthy controls, or drug discovery trials, focussing on dose reduction for the accompanying CT scan is a key task.

### 1.1 CT dosimetry

The radiation dose delivered to a patient via the absorption of the X‐rays generated by the CT depends on the scanner hardware and configuration as well as software to modulate the CT settings. A CT acquisition is typically characterized by the applied tube voltage $U_{\mathrm{tube}}$ and current $I_{\mathrm{tube}}$, the exposure time, and the pitch. Possible ways for dose reduction are decreasing the tube voltage or current, reducing the rotation time, and increasing the pitch. Since the pitch possibly impacts the CT spatial resolution and image quality,^14^, ^15^ this study will focus on the reduction of tube voltage and exposure and the usage of tin (Sn) filters to reshape the CT spectrum and avoid changing any geometrical components. It has to be kept in mind that using a low pitch can potentially increase the impact of respiratory motion on the CT images.

#### 1.1.1 Exposure and tin filtration

The tube voltage and current determine the energy and number of γ‐photons emitted by the CT. Increasing those parameters will result in an increased radiation dose $D_{\mathrm{rad}}$ delivered to the patient. This increase is linear with the tube current $I_{\mathrm{tube}}$ and approximately follows a squared relation with the tube voltage $U_{\mathrm{tube}}$ ^16^

(1) $$D_{\mathrm{rad}}\propto U_{\mathrm{tube}}^{2}\cdot I_{\mathrm{tube}}$$

The tube voltage and current can be modulated based on the patient size. Commercial CT scanners use the scout scan or “topogram” as input to vary the tube voltage and current based on the patient diameter and density in each slice, resulting in higher voltages and currents being delivered at larger diameters and higher densities such that the image quality for a specified reference tube voltage and current is maintained.

Tin filters can be employed to attenuate the lower energies of the CT X‐ray spectrum,^17^ which would otherwise contribute to additional dose to the patient. It has been demonstrated that this spectral shaping has limited impact on image quality for different diagnostic tasks and anatomical regions^18^, ^19^ and may help increase image contrast in specific imaging and reconstruction protocols.^20^ The tin filter leads to a hardened CT beam and calls for adjustments in the associated dose conversion factors (see Section 1.1.2 and Equation 2).^21^ Additionally, the entire photon spectrum is heavily attenuated, calling for higher tube voltage and exposure compared to non‐Sn filtered spectra.

#### 1.1.2 Effective dose

The radiation dose to the patient is typically characterized as the effective dose (ED). Empirical conversion factors $k_{i}$ ^22^ convert the region‐specific dose length product (DLP) to regional effective dose ${\mathrm{ED}}_{i}$ ^23^

(2) $${\mathrm{ED}}_{i}={\mathrm{DLP}}_{i}\cdot k_{i}$$

The factors $k_{i}$ account for different attenuation properties in regions such as the abdomen, head, neck, chest, proximal thigh, and pelvis.

### 1.2 Purpose of this study

It is expected that reducing tube voltage and exposure as well as the use of tin filters will impact the CT beam profile and cause slight differences in the registered HU, which might not be the same for different types of tissue.^24^, ^25^, ^26^, ^27^, ^28^ In addition, structures of high density, such as bones, can result in beam hardening and photon starvation depending on the selected CT protocol. Reducing the CT dose must therefore be carefully balanced with any impact on the CT image quality and quantitative metrics, which can potentially be transferred to the PET image via attenuation correction.

The dimensions of standard PET quantification phantoms (e.g., NEMA‐IQ) are small compared to the body of an adult patient and usually water‐based and so exhibit insufficient density, structural variation and thickness to properly assess the attenuation of γ‐photons in the human body. This also results in a lack of material‐specific HU differences and any structures to produce significant artefacts in a CT image, which are important in asssessing the accuracy and quantitative impact of the attenuation correction applied to a PET image. This study presents a custom phantom assembly scanned on the Biograph Vision Quadra (Siemens Healthineers) which allows quantification assessments in soft tissue and bone and in regions with beam hardening and photon starvation (as is expected in an arms‐down scanning protocol). The phantom also allows assessment of the effects of material‐specific HU differences. Existing literature does not map out the large parameter space of this study^29^, ^30^, ^31^, ^32^ and lacks the evaluation of potential artefacts in arms‐down scans. The accuracy of attenuation and scatter correction via the lung residual error and the impact of including a TOF kernel in the reconstruction have not been quantified before.^29^, ^30^, ^31^, ^32^

We refrain from the use of dose modulation, which was applied in previous studies,^29^, ^32^ as this hinders the evaluation of the dose savings for targeting specific applications. Precisely controlling radiation exposure with fixed‐parameter protocols is key in these cases. Especially for imaging pregnant patients, fixed‐parameter CT protocols are of utmost importance to prevent any harm to the foetus. Dose‐modulated CT protocols largely adjust their dose depending on the object thickness in a slice, leading to increased radiation exposure to the womb of a pregnant patient, that is, the foetus. In the following, we

‐ assess the potential reduction in CT radiation dose arising from the use of tin filtration,
‐ analyze differences in CT quantitative metrics, that is, HU and noise, and report the presence of artefacts,
‐ extensively evaluate differences in PET quantification, that is, SUV, SNR, lung residual error and background variability,

and provide a discussion of the impact of the results on the choice of clinical protocol.

## 2 MATERIALS AND METHODS

The impact of different CT parameters on PET quantification, when using the CTs for attenuation correction and scatter estimation, was assessed by acquiring scans of a custom NEMA‐IQ phantom on a Biograph Vision Quadra (Siemens Healthineers, Knoxville, TN, USA). The scanner features a 128‐slice SOMATOM^®^ Definition Edge CT scanner.

### 2.1 Phantom preparation

The NEMA‐IQ phantom was prepared and filled with [^18^F]FDG in a 5‐to‐1 activity concentration ratio (actual measured ratio 5.4‐to‐1), resulting in a total activity of about 62MBq at the time of the PET scan. The phantom was placed on the bed and covered with saline bags (28mmto87mm thickness around the phantom, see Figure 1) to imitate soft tissue around the abdomen. The resulting dimensions of the phantom (max. diameter 47cm) cover typical patient diameters for a range of body mass indices.^33^ Sealed bovine marrow bones (bovine long bones, diameter of approx. 50mm, see Figure 1), bought from a store which sells for human consumption, were placed left and right of the phantom, at the same height, to resemble the humerus or elbows of a patient being scanned with arms down. Other similar cases include the shoulder joints in lung imaging or the hip joints in prostate imaging. Dense structures on either side of the phantom are likely to produce streak artefacts and photon‐starved regions. Their high density results in a high number of photons being absorbed by the structures, ultimately starving the CT detector and leading to dark, band‐like artefacts spanning the images. At the same time, again by their high absorption, denser structures are more likely to increase statistical noise as fewer photons reach the detector. Two vials with diameter of 10mm each, one filled with [^18^F]FDG and saline solution, and one filled with sand, [^18^F]FDG and saline solution, were placed inside the sealed marrow bones to mimic a lesion in soft tissue close to bone or within bone (see Figure 1). We validated that the use of sand results in similar HU for the vial background as in bone for all acquired CTs^30^ enabling the assessment of quantification errors in bone structures. The activity concentration used to fill these vials was the same as for the spheres in the NEMA‐IQ phantom. Part of the NEMA‐IQ phantom was intentionally left uncovered so that the background variability could be evaluated with and without attenuation from the saline bags and bones, that is, with and without the presence of artefacts produced by the surrounding materials. A “lung” insert containing small Styrofoam pearls was inserted in the center of the phantom to assess scatter. The assembly was placed well within the transaxial fields of view for both PET and CT to rule out any edge effects. We placed five 50‐ml Falcon^™^ tubes (diameter of 30mm) on the bed, each of them filled with a different material to monitor any material‐specific HU differences for all acquired CTs. The tubes contained water, dry flour, flour mixed with water, dry sand, and sand mixed with water.^30^ Furthermore, a sealed bovine marrow bone was placed on the bed to investigate any bone‐specific HU differences. Figure 2 depicts the phantom assembly on the scanner bed.

**FIGURE 1:**
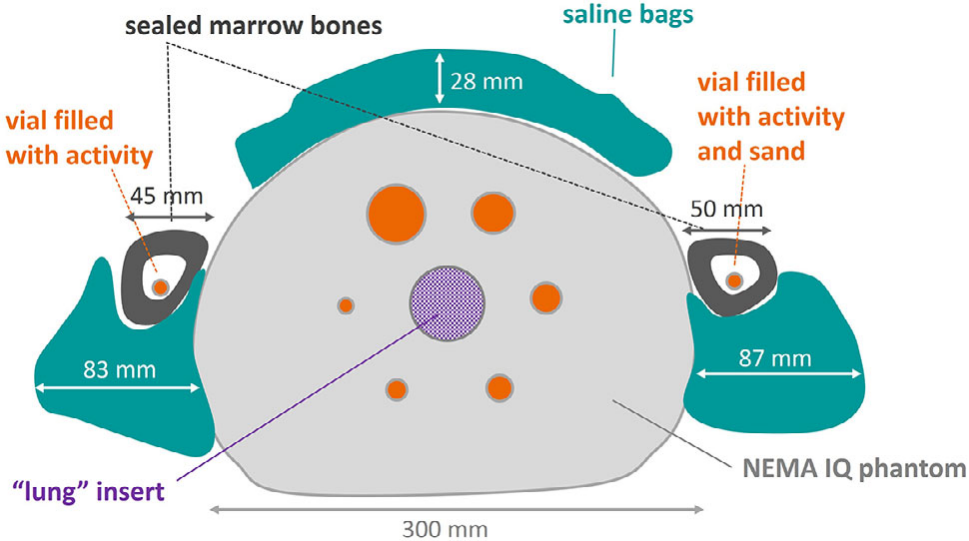
Sketch of the NEMA‐IQ phantom assembly. The saline bags are used to resemble soft tissue, while the sealed marrow bones simulate the arms of a patient. Two vials were placed inside the marrow bones to mimic a bone metastasis.

**FIGURE 2:**
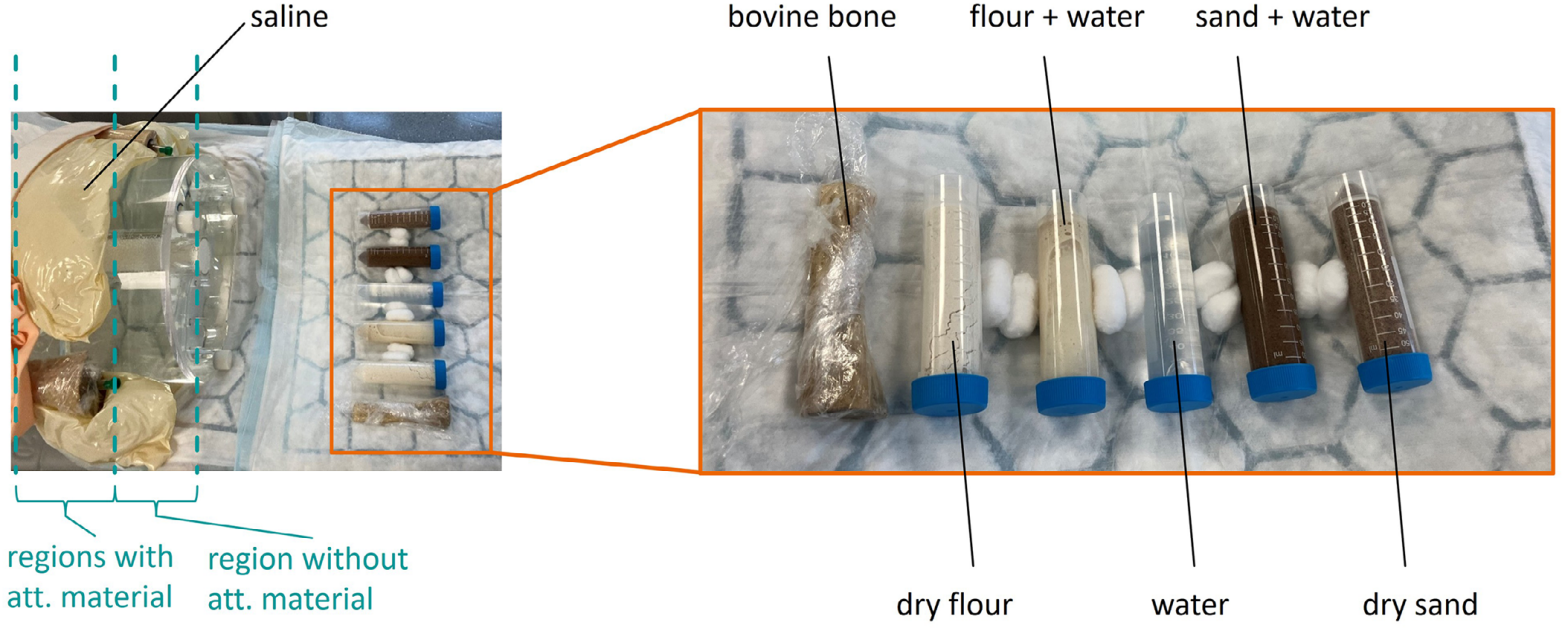
Custom phantom assembly on the bed. Next to the NEMA‐IQ phantom, samples of different materials were scanned to monitor any material‐specific HU differences.

### 2.2 Protocol and reconstruction

#### 2.2.1 CT protocol and reconstruction

In total, 47 CTs were acquired (single acquisitions with different tube voltage and exposure settings). CTs with tube voltages of 100kV, 120kV, and 140kV without tin filtration and 100kV and 140kV with tin filtration (denoted as Sn‐100kV and Sn‐140kV) were acquired. For each voltage, the exposure was set to 7mAs, 13mAs, 25mAs, 30mAs, 50mAs, 100mAs, 200mAs, 300mAs, and 400mAs. An exposure of 7mAs is considered the minimum exposure configuration according to factory recommendations, while the tube current is limited to 20mA. To test the HU change in ultra‐low‐dose CT protocols, two scans were acquired with 70kV and 100mAs as well 80kV and 50mAs, both resulting in a similar dose. CTs were acquired with a fixed rotation time of 0.5s, a fixed pitch of 0.8 and a scan duration of 18.09s. The collimation was set to 128× 0.6mm. To permit scanning with an exposure of 7mAs, the scan duration was reduced to 8.93s for all tube voltages. To permit scanning with an exposure of 400mAs, the rotation time was increased to 1.0s for all tube voltages, resulting in an increased scan duration of 36.17s. All acquired CTs were reconstructed using a Br38f kernel and filtered back projection with an image size of 512×512 pixels per slice and a slice thickness of 3mm. The settings 120kV and 50mAs were considered as a typical clinical whole‐body CT protocol and as a reference point to evaluate any differences. This protocol is considered to provide accurate attenuation correction (i.e., not of the quality of a diagnostic stand‐alone CT). Localisation and diagnostic quality of the CT are not evaluated in this study, which both require a clinical assessment. Tube current modulation (CareDose4D; Siemens Healthineers^34^) and tube voltage modulation (CarekV; Siemens Healthineers^34^) were disabled for all scans to ensure scanning with a fixed protocol. Since this study focuses on phantom scans, dose modulation was not considered to provide any additional insights as, compared to a patient, the phantom is quite uniform and small, resulting in the same effective voltage and exposure being configured for a wide range of settings, and therefore does not permit a thorough evaluation of CT parameter range despite additional potential dose savings.

#### 2.2.2 PET protocol and reconstruction

A ten‐minute static PET scan was acquired using a clinical half‐body protocol (106cm aFOV). The PET images were reconstructed with an image size of 440×440 pixels per slice, resulting in 1.65mm × 1.65mm × 1.65mm voxels. An iterative ordered‐subset estimation maximization (OSEM) reconstruction algorithm (4 iterations, 5 subsets) was used in ultra‐high sensitivity (UHS) mode (maximum ring difference 322). A spatially variant point spread function (PSF)^35^ with an axial width of 1.5mm was modeled in the reconstruction. Sinograms were binned into 33 TOF bins with a width of 143ps.^36^ No Gaussian smoothing was applied. Each of the different CTs was used for attenuation and scatter estimation (see Section 2.2.3), resulting in 47 different PET reconstructions. For all offline reconstructions, we used the reconstruction within e7tools software (Siemens Healthineers).

#### 2.2.3 Generation of attenuation map and scatter estimation

An attenuation map was generated for each of the acquired CT protocols by smoothing the CT images with a Gaussian kernel and converting the Hounsfield units (HU) to attenuation correction factors at 511 keV using a bilinear model.^24^, ^27^, ^28^ The model constants applied were specific to the tube voltage and accounted for the use (or not) of a tin filter. The generated attenuation map was down‐sampled to the PET voxel size, and scatter was estimated via a single scatter simulation.^37^, ^38^, ^39^, ^40^ Overall, attenuation correction, scatter correction, normalization and corrections for randoms, deadtime and decay time were applied to the PET images. All correction methods applied were implemented with e7tools (Siemens Healthineers).

### 2.3 Evaluation metrics

#### 2.3.1 Radiation dose assessment

To assess the impact of different CT protocols on the radiation dose delivered, the DLP as reported in the scanner's dose report was used. As the size of the phantom used in this study is smaller than patients, it is difficult to estimate accurate dose conversion factors as suggested in Equation 2, especially if the dose assessment is aimed at different body regions.

#### 2.3.2 CT image quality and quantitative analysis

##### Visual assessment

CT images were visually compared and checked for beam hardening artefacts, which were expected to be caused by the two bones placed laterally to the phantom. If artefacts were present, their localization with respect to the six NEMA‐IQ spheres was assessed to analyze effects seen in quantitative PET metrics with respect to streaks seen in the CT.

##### CT image noise in water

As an estimate of CT image quality, the noise in the uniform background of the phantom was measured by drawing six circular regions of interest (ROIs, diameter 6 cm) in five slices, resulting in 30 regions in total. The mean standard deviation (${\bar{\sigma }}_{\mathrm{HU}}$) was computed from the individual standard deviation of the HU in these ROIs $\sigma_{\mathrm{HU}},i$ as a measure for the average CT image noise in water

(3) $${\bar{\sigma }}_{\mathrm{HU}}=\frac{1}{N}\sum_{i}^{N}\sigma_{\mathrm{HU}},i$$

This procedure was repeated for five slices outside of the sphere environment, that is, a phantom region where no attenuating material was placed (see Figure 2), to quantify the impact of a larger object and higher attenuation on the CT image noise.

##### Material‐specific HU difference

Changing the tube voltage and using tin filtration are expected to have an impact on the material‐specific HU in the acquired CT image due to differences in the CT energy spectrum. CTs with different tube voltages (70kV, 80kV, 100kV, 120kV, 140kV, Sn‐100kV, and Sn‐140kV) were evaluated. The exposure is not expected to impact this assessment, so it was kept constant at 50mAs, except for the scan with 70kV, which was acquired with an exposure of 100mAs, to give a similar DLP as the 80kV protocol. To quantify the material‐specific HU, one circular ROI (diameter 2.25mm) was drawn in each of the non‐osseous samples (water, saline, dry flour, flour mixed with water, sand, and sand mixed with water). The mean HUs as well as their standard deviations were computed from this ROI. As the shape of the bovine bone sample does not permit the use of a circular ROI with a defined radius, a slice displaying the bovine bone sample was segmented with a threshold of 600HU in this slice. The mean and its standard deviation were computed from this segmented ROI.

#### 2.3.3 PET image quality and quantitative analysis

##### Visual assessment

The 47 reconstructed PET images were visually compared. Attention was paid to whether all six spheres were resolved and if there was any trace of CT artefacts being transferred to the PET image via the attenuation map. Difference images with respect to the image reconstructed with the typical clinical CT (120kV, 50mAs) were computed to aid this assessment.

##### Sphere and vial localization

The center of each of the six NEMA‐IQ spheres was localized by computing the line and gradient profiles of the spheres along each image axis. A peak finder was applied to identify peaks in the gradient profiles. The mean of these peaks was used to determine the central coordinate of the sphere on each axis and the distance between the peaks was taken as the width of the sphere. The same process was applied to identify the center of both vials that contained activity. The ROIs of the NEMA‐IQ spheres and the vials were identified for the typical clinical protocol (120kV, 50mAs) images and applied to all reconstructed images. Fixed ROIs are used to avoid additional contributions that might affect the quantification assessment. The NEMA‐IQ spheres have very well defined and sharp edges, hence we expect any effects of not delineating ROIs on individual images to be negligible.

##### Standardized uptake values in NEMA‐IQ spheres

Using the coordinates and diameters for the spheres, the standardized uptake value (${\mathrm{SUV}}_{\mathrm{mean}}$) was computed for the masked region from the mean activity concentration in the respective ROI.

##### Standardized uptake values in vials representative of bone metastasis

A spherical ROI with the same diameter as the smallest sphere was placed in the center of each vial and the ${\mathrm{SUV}}_{\mathrm{mean}}$ was computed for these ROIs.

##### Background variability

The background variability (BVr), for each sphere diameter, was computed as the standard deviation $\sigma_{\mathrm{ROI}}$ of the mean value $\mu_{\mathrm{ROI}},i$ in 60 individual ROIs (five slices with twelve regions per slice) with respect to the overall mean value ${\bar{\mu }}_{\mathrm{ROI}}$.^41^

(4) $${\mathrm{BV}}_{r}=\frac{\sigma_{\mathrm{ROI}}}{{\bar{\mu }}_{\mathrm{ROI}}}=\frac{\sqrt{\frac{1}{N-1}\sum_{i}^{N}{\left(\mu_{\mathrm{ROI},i}-{\bar{\mu }}_{\mathrm{ROI}}\right)}^{2}}}{{\bar{\mu }}_{\mathrm{ROI}}}$$

The ROI size was varied to match the diameter of the spheres. This computation was applied to five slices in a region surrounded by the saline bags and bones and to five slices in a region without surrounding attenuating materials. Selecting two different regions helped assess the impact of higher attenuation coefficients on the background of the PET image.

##### PET signal‐to‐noise ratio

The PET SNR is given by

(5) $${\mathrm{SNR}}_{i}=\frac{{\bar{C}}_{i}-{\bar{C}}_{\mathrm{bkg}}}{\sigma_{\mathrm{bkg}}}$$

which is the difference between the mean activity concentration in a sphere ${\bar{C}}_{i}$ (i=1…6) and the background ${\bar{C}}_{\mathrm{bkg}}$, divided by the standard deviation of the mean activity concentration in the background $\sigma_{\mathrm{bkg}}$ for the largest ROI selected.

##### Accuracy of attenuation correction and scatter estimation

To assess the accuracy of the attenuation correction and scatter estimation, the reconstructed activity concentration within the volume of the central “lung” insert of the NEMA‐IQ phantom was evaluated. The “lung” residual error $\Delta C_{\mathrm{lung}}$ is then given by the ratio of the mean activity concentration in the ”lung” insert versus the background activity concentration ${\bar{C}}_{\mathrm{bkg}}$ ^42^

(6) $$\Delta C_{\mathrm{lung}}=100\cdot \frac{{\bar{C}}_{\mathrm{lung}}}{{\bar{C}}_{\mathrm{bkg}}}$$

The factor 100 accounts for a representation in %.

## 3 RESULTS

### 3.1 Radiation dose assessment

Figure 3 depicts the DLP recorded for each protocol, which decreases linearly with the exposure. Table 1 reports the corresponding CTDIvol for each protocol. The highest DLP reduction with the acquired protocols is about 99% for the protocol with a tube voltage of Sn‐100kV and an exposure of 7mAs compared to the typical clinical protocol (120kV, 50mAs). Without using a tin filter and only by reducing the tube current, the DLP reduction is about 80% for the protocol with a tube voltage of 120kV and an exposure of 7mAs compared to the typical clinical protocol. As reported in Section 1.1.2, the DLP solely depends on the CT protocol and scanner‐specific parameters and does not assess the patient‐specific effective radiation dose. However, a relative reduction in DLP will translate to a reduction in patient dose.

**TABLE 1:** CTDIvol in mGy as function of the exposure (mAs) and tube voltage (kV; no current or voltage modulation applied). The length of the phantom was 24cm.

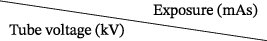	400 mAs	300 mAs	200 mAs	100 mAs	50 mAs	30 mAs	25 mA	13 mAs	7 mAs
140 kV	40.88	30.53	20.44	10.28	5.14	3.09	2.57	1.41	0.78
120 kV	26.79	20.01	13.39	6.70	3.37	2.02	1.68	0.88	0.51
100 kV	15.58	11.67	7.83	3.91	1.97	1.18	0.98	0.52	0.30
Sn‐140 kV	8.91	6.67	4.46	2.23	1.12	0.67	0.56	0.29	0.17
Sn‐100 kV	1.60	1.20	0.80	0.40	0.20	0.12	0.10	0.05	0.01

**FIGURE 3:**
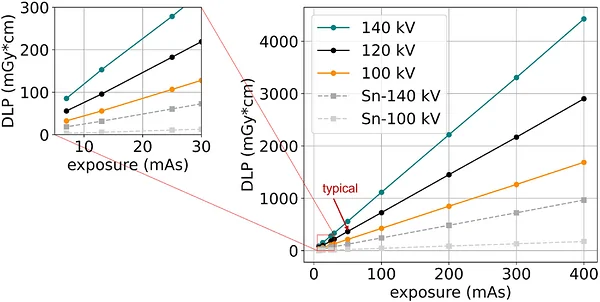
DLP as function of the exposure (no current or voltage modulation applied). The DLP of the typical clinical protocol (120kV, 50mAs) is 365mGycm.

### 3.2 CT image quality and quantitative analysis

#### 3.2.1 Visual assessment

We compiled a grid of CT images of the same phantom slice with 45 different CT protocols to visually assess the impact of the different settings (see Figure 4). All phantom structures including saline bags, bones and vials in bones are visible in all CT images. The outer contour of the NEMA‐IQ spheres is visible for high tube voltages and currents but becomes more difficult to see for lower tube voltages and currents. At constant tube voltage, the images become slightly noisier with decreasing exposure, which is quantitatively assessed in Section 3.2.1. We observe streak artefacts for 140kV at 13mAs and below, for 120kV at 25mAs and below, for Sn‐140kV at exposures of 30mAs and below, and for 100kV and Sn‐100kV at all exposures. The intensity of the artefacts increases with decreasing exposure. Additionally, we observe regions of photon starvation for 120kV at 13mAs and 7mAs, for 100kV for exposures of 50mAs and below, and for Sn‐100kV for exposures of 200mAs and below.

**FIGURE 4:**
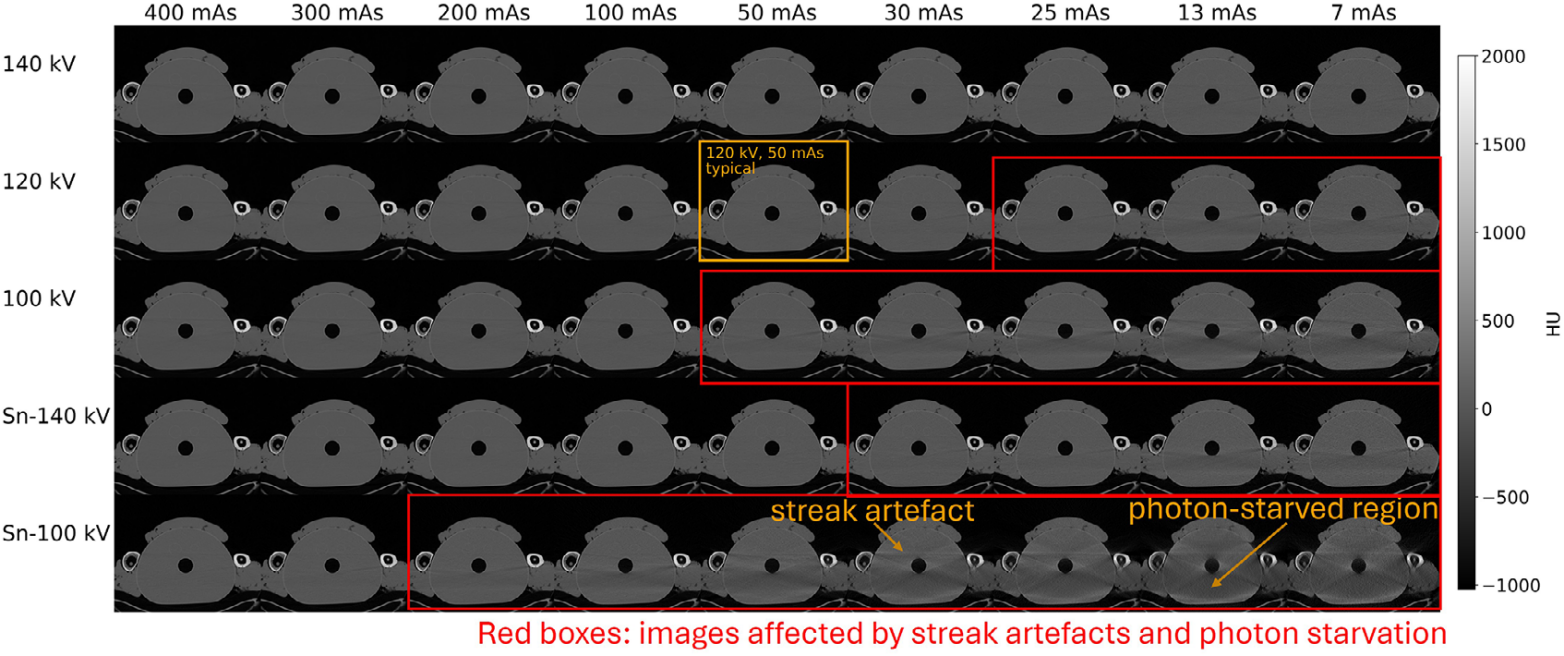
Reconstructed CT images for 45 different protocols. The image for the typical clinical protocol (120kV, 50mAs) is highlighted by an orange frame. The displayed axial slice is from the phantom region surrounded by attenuating material, see Figure 2.

#### 3.2.2 CT image variability in water

The CT images of the NEMA‐IQ phantom show an average standard deviation of 5HUto10HU in the phantom background for exposures of 100mAs or larger and all tube voltages except Sn‐100kV. Configuring a tube voltage of Sn‐100kV results in a constantly higher noise for all exposures. The observed effects are the same for slices in a region without attenuating materials surrounding the phantom with no artefacts produced (see Figure 5 (left)) and for slices in a region of the phantom covered by saline bags and bones, where streak artefacts and photon‐starved regions were produced (see Figure 5 (right)). In both cases, the standard deviation increases exponentially with a reduction of the exposure for all tube voltages. The increase in standard deviation is larger in regions where artefacts are produced by surrounding attenuating materials.

**FIGURE 5:**
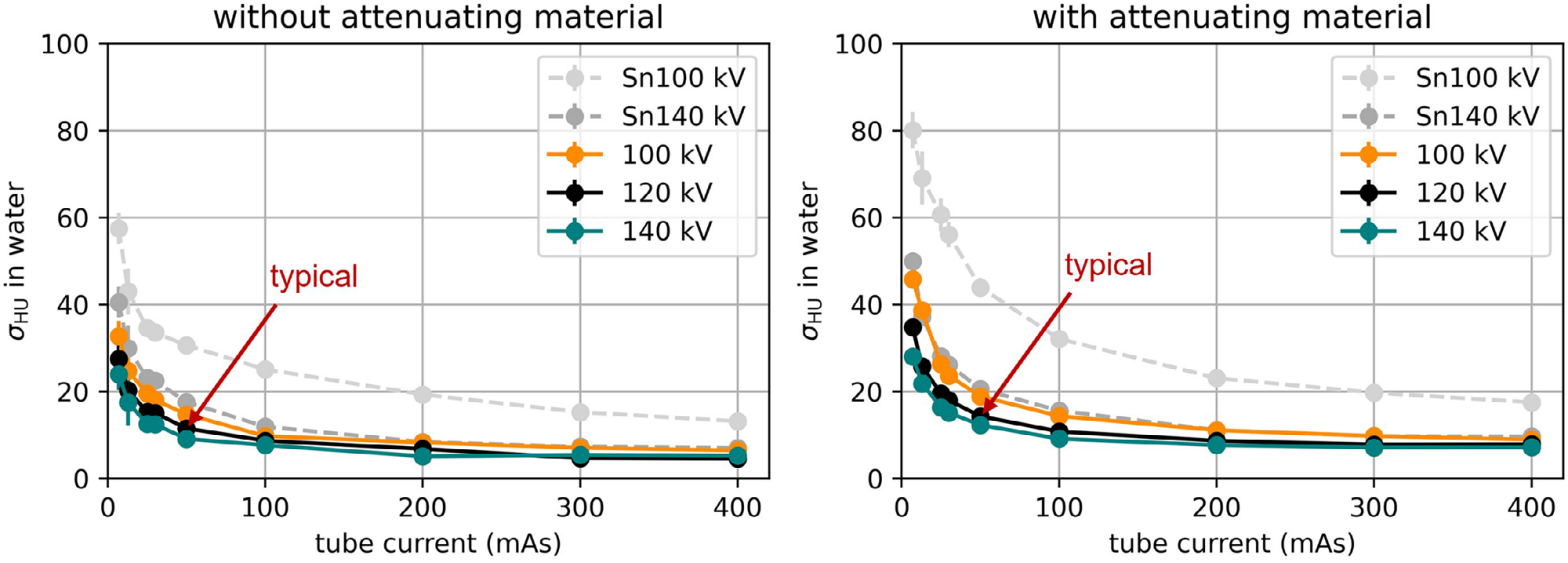
Standard deviation of the HU in water for 30 ROIs in a region without attenuating materials with no artefacts (left) and in a region covered by saline bags and bones producing streak artefacts and photon‐starved regions (right).

#### 3.2.3 Material‐specific HU differences

The material‐specific HU changed depending on the tube voltage of the CT protocol. CT attenuation values for human cortical bone vary according to anatomical site and imaging protocol but are commonly reported in the range of approximately 500HU to 900HU,^43^ with values > 1000HU also described.^44^ The bovine cortical bone in our phantom demonstrated attenuation values of 900HU to 1500HU, not dissimilar to the range expected for human cortical bone. Although the dimensions of bovine bones may be larger than human bones, such as the humerus, the attenuation properties provide a reasonable cortical bone surrogate. Trabecular bone is more porous and less dense, with reported attenuation values around 85HU and 200HU,^45^ although much higher values^46^ may result depending on anatomical site, trabecular density, marrow composition and acquisition protocol. The use of bovine bone together with a sand‐water mixture in our custom NEMA‐IQ phantom provides a pragmatic method for reproducing a broad range of bone‐equivalent attenuation values spanning cortical and trabecular bone.

In the following, we discuss the absolute differences compared to a protocol with a tube voltage of 120kV. HU differences and noise (indicated by the error bars showing the standard deviation of the values in the chosen ROI) were greater in bone (50HUto500HU difference) than in other materials (less than 10HU difference). Furthermore, as exemplarily displayed in the lower plot in Figure 6 for the sand‐water mixture, the HU did not change for different exposures at constant tube voltage (less than 10HU difference). The noise was larger in water, saline and flour (> 100% relative to the registered HU) and smaller in bone and sand (approximately 5%to30%). It is evident that both sand and sand mixed with water exhibit similar differences and trends to bone, which is why we consider sand mixed with [^18^F]FDG and saline solution to be a suitable surrogate to assess the impact of CT protocols on the PET quantification in bone, especially if comparing the impact relatively to a typical clinical protocol.

**FIGURE 6:**
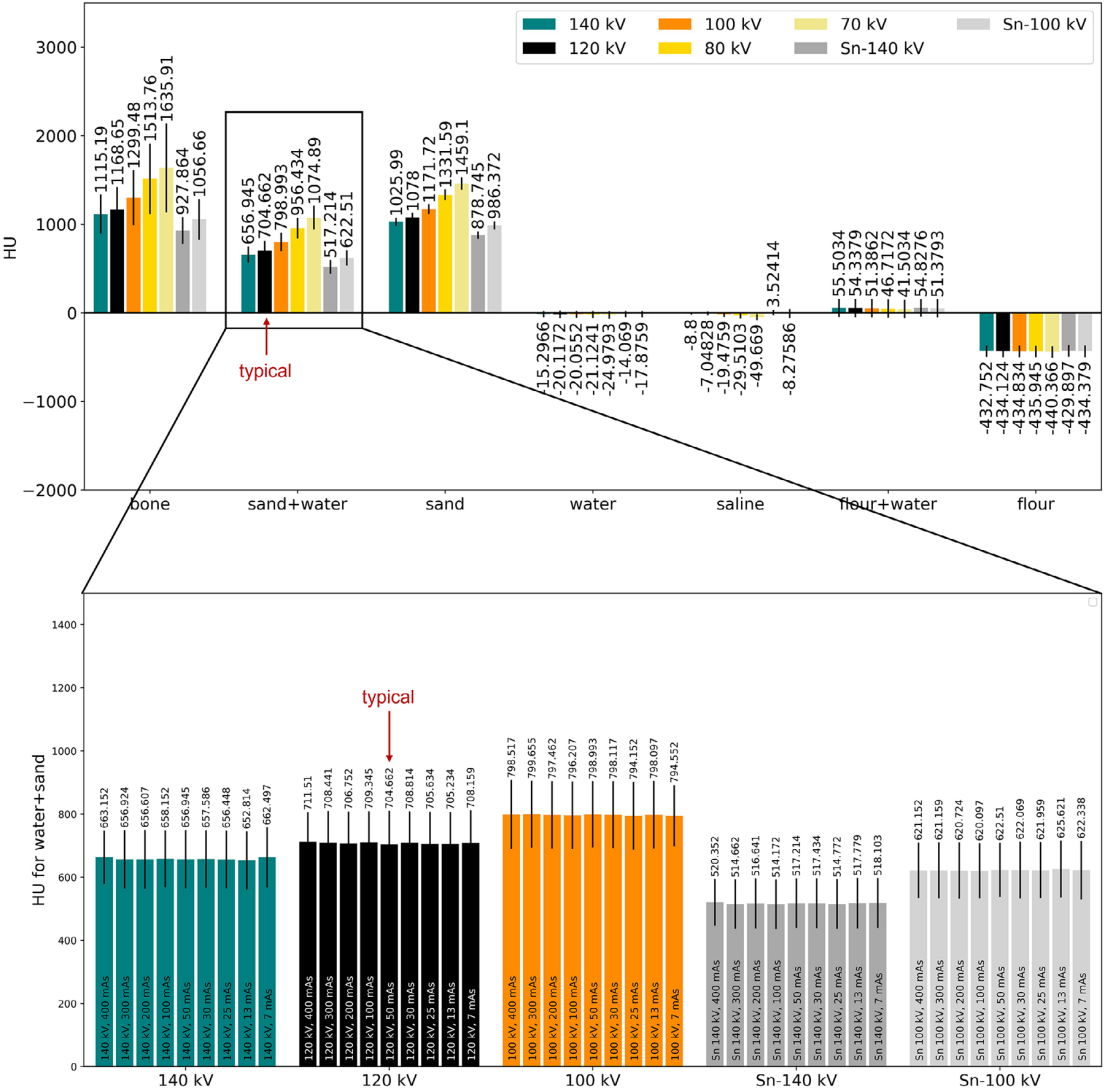
Material‐specific HU differences across different tube voltage settings (top) and for different exposures of the sand‐water mixture (bottom).

### 3.3 PET quantification

#### 3.3.1 Visual assessment

A PET image with attenuation map extracted from one of the CT images is shown for an example slice of the phantom at Sn‐100kV and 30mAs in Figure 7. It becomes evident that the artefacts seen for the CT images in Figure 4 are transferred to the attenuation map and that their location affects the NEMA‐IQ spheres differently. For example, the smallest NEMA‐IQ sphere (10mm) overlaps with a streak artefact, while the NEMA‐IQ spheres with 13mm and 17mm diameter are localized in a region suffering from photon starvation due to the thick layers of saline left and right of the phantom. Figure 8 shows the PET images reconstructed with CT images from 45 different protocols used for attenuation correction and scatter estimation. All PET images resolve the six NEMA‐IQ spheres (diameters 10mm, 13mm, 17mm, 22mm, 28mm. and 37mm). Visually, the images do not show noticeable differences, except for a slight decrease in contrast for a tube voltage of Sn‐100kV combined with exposures of 13mAs and 7mAs. To further investigate the transfer of artefacts to the PET image, we computed difference images with respect to the PET images reconstructed with the typical clinical protocol, which are shown in Figure 9.

**FIGURE 7:**
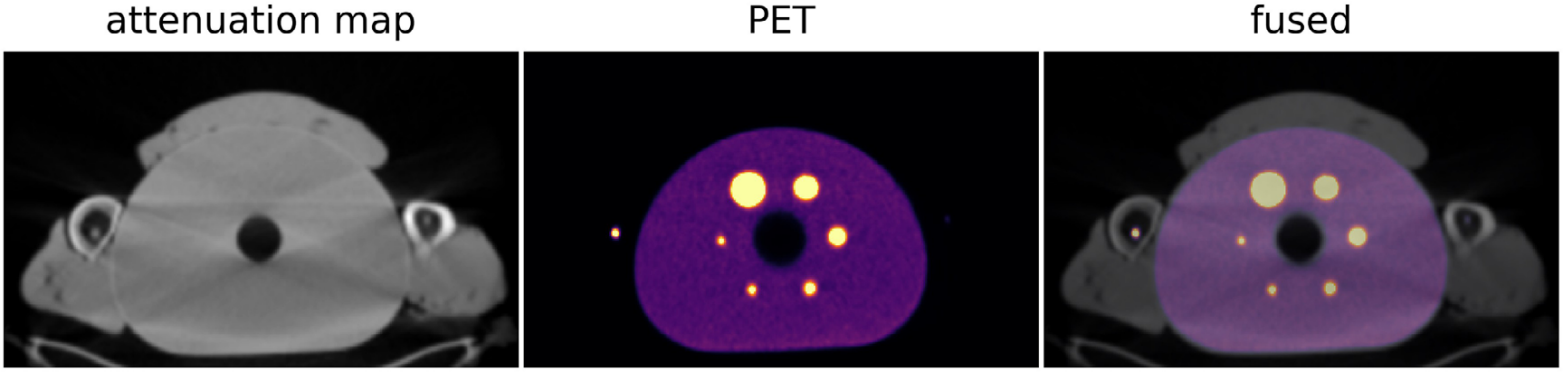
Reconstructed PET image with the attenuation map generated from a CT with Sn‐100kV and 30mAs. Left to right: Attenuation map, PET image, fused PET image and attenuation map.

**FIGURE 8:**
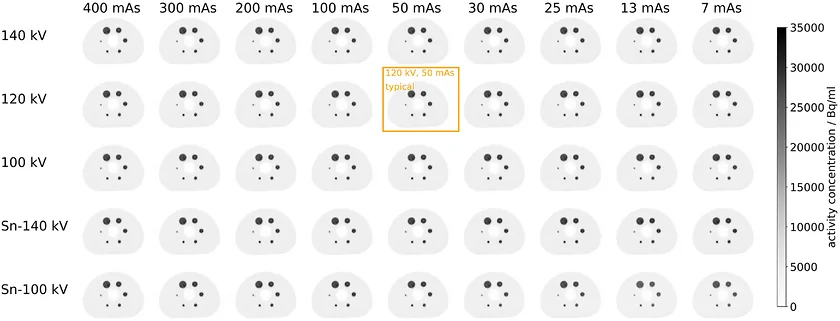
Reconstructed PET images using 45 different CT protocols for attenuation correction and scatter estimation. The image for the typical clinical protocol (120kV, 50mAs) is highlighted by an orange frame.

**FIGURE 9:**
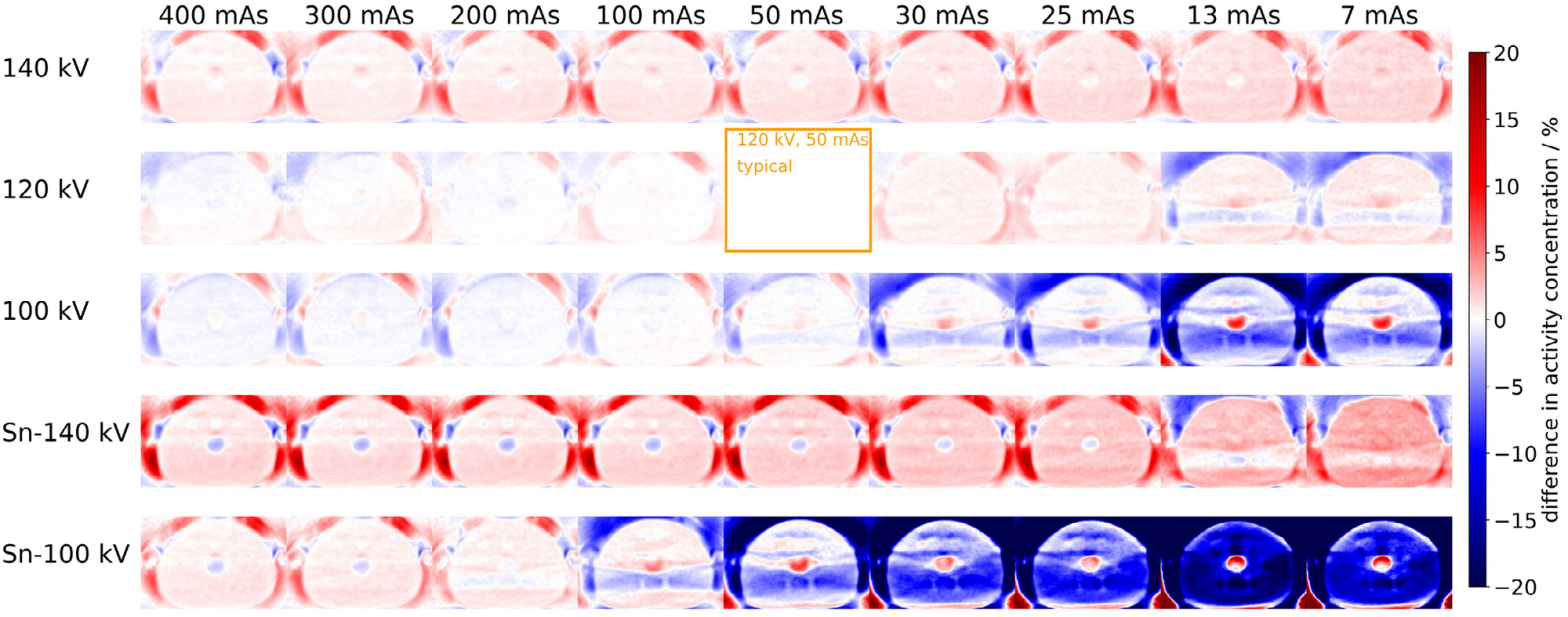
PET difference images compared to the image reconstructed with the typical clinical CT (120kV, 50mAs). The color bar shows the relative difference in activity concentration and is saturated at ± 20%. The image for the typical clinical protocol (120kV, 50mAs) is highlighted by an orange frame.

Configuring a tube voltage of 140kV and Sn‐140kV leads to a slight elevation in measured activity concentration in the phantom (< 5% difference), while a tube voltage of 100kV results in a decrease (< 5% difference) compared to the typical clinical protocol. Streak artefacts are visible in almost all difference images. Images reconstructed with 120kV and 13mAs. and 7mAs; 100kV and exposures of 50mAs and below; and Sn‐100kV and exposures of 100mAs and below, show the photon‐starved region in the lower part of the phantom, which is associated with a decrease in activity concentration of up to 20% and more.

#### 3.3.2 Standardized uptake values in NEMA‐IQ spheres

Recovery curves based on the ${\mathrm{SUV}}_{\mathrm{mean}}$ show similar behavior at all tube voltages with and without tin filtration and slightly overshoot the expected value of 5 based on the 5.4‐to‐1 filling ratio. At a tube voltage of Sn‐100kV, the impact of the exposure on the measured ${\mathrm{SUV}}_{\mathrm{mean}}$ becomes apparent. Here, higher exposures maintain the recovery of the ${\mathrm{SUV}}_{\mathrm{mean}}$, while lower exposures are associated with higher noise and artefacts and reduce the measured ${\mathrm{SUV}}_{\mathrm{mean}}$ by approximately 10% (sphere diameters 10mm and 13mm) to 20% (sphere diameters 17mm and above). For most protocols, despite the display of minor artefacts and regions with slight photon starvation, the measured ${\mathrm{SUV}}_{\mathrm{mean}}$ remains within 2% of the value measured with the typical clinical protocol. The threshold of 2% encompasses roughly 90% of the protocols. The only exception (approx. 3%to17.5% difference depending on sphere size) are tin‐filter protocols with Sn‐140kV and an exposure of 7mAs and 13mAs, and Sn‐100kV and exposures of 30mAs and below for all sphere sizes. The ${\mathrm{SUV}}_{\mathrm{mean}}$ of the spheres of 13mm and 17mm diameter, which are localized in the CT photon‐starved region of the NEMA‐IQ phantom, also shows larger deviations for the protocols with 100kV and an exposure of 7mAs and 13mAs.

#### 3.3.3 Standardized uptake values in vials mimicking bone metastasis

To assess the accuracy of measured ${\mathrm{SUV}}_{\mathrm{mean}}$ in regimes with different HU, specifically in bone, we looked at the relative ${\mathrm{SUV}}_{\mathrm{mean}}$ in the two vials, one filled with [^18^F]FDG and saline solution and one filled with [^18^F]FDG and saline solution mixed with sand, positioned inside the two marrow bones left and right of the NEMA‐IQ phantom and compared the behavior of the ${\mathrm{SUV}}_{\mathrm{mean}}$ difference to the effects seen in the smallest NEMA‐IQ sphere (diameter 10mm, same as the vials). For the vial filled with activity, mimicking soft tissue or vessels close to bone, the difference in ${\mathrm{SUV}}_{\mathrm{mean}}$ remained within 2% of the value obtained with the typical clinical CT protocol (120kV, 50mAs) except for protocols with Sn‐100kV and exposures of 30mAs and[Fig mp70706-fig-0010] below (see Figure 11). For the vial filled with sand and activity, mimicking a bone metastasis, the difference in ${\mathrm{SUV}}_{\mathrm{mean}}$ remained within 2% of the value obtained with the typical clinical CT protocol (120kV, 50mAs). Here, the protocols exceeding the 2%‐range are Sn‐140kV with an exposure of 7mAs and Sn‐100kV with exposures of 25mAs and below (see Figure 11).

**FIGURE 10 mp70706-fig-0010:**
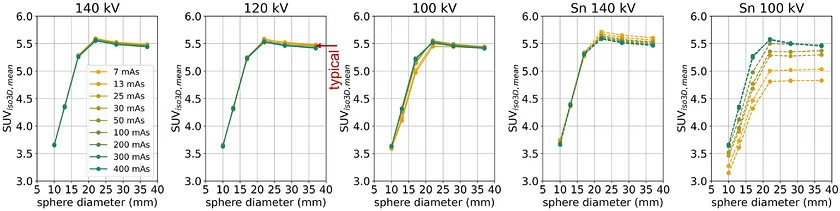
Recovery curves for different tube voltages and currents.

**FIGURE 11:**
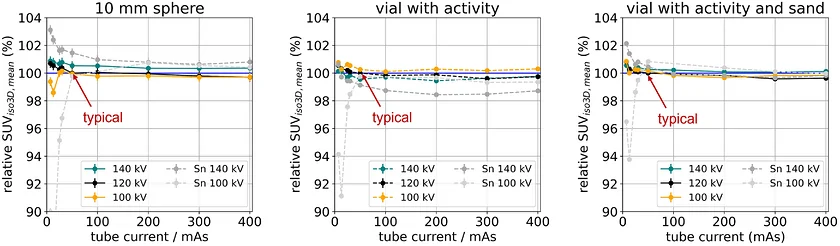
Relative differences of the ${\mathrm{SUV}}_{\mathrm{mean}}$ in the 10mm sphere (left image), the vial filled with activity (middle image) and the vial filled with activity and sand (right image) for different tube voltages and currents compared to the typical clinical protocol with 120kV and 50mAs.

#### 3.3.4 Background variability

The ${\mathrm{BV}}_{r}$ in the NEMA‐IQ phantom increased due to the presence of attenuating material (saline bags and bones) and related artefacts (photon starvation and streak artefacts). Figure 12 shows this increase (colored curves) in comparison to slices of the phantom that were intentionally left free of attenuating material (grey curves). The ${\mathrm{BV}}_{r}$ in the region with higher attenuation was between 2% and 4% for all tube voltages and currents. In the region with lower attenuation and in absence of artefacts, the ${\mathrm{BV}}_{r}$ decreased to 1%to2.25% (see Figure 12). For lower tube voltages, for example, 100kV and Sn‐100kV, the exposure had an impact on the ${\mathrm{BV}}_{r}$, with reduced exposures leading to an increased ${\mathrm{BV}}_{r}$. For most protocols and all ROI sizes, the difference in ${\mathrm{BV}}_{r}$ remained below 10%. Exceptions were protocols with tube voltages of Sn‐100kV for exposures of 100mAs and below, and tube voltages of 100kV with exposures of 13mAs and 7mAs. The smaller the size of the selected ROI, the larger the increase in ${\mathrm{BV}}_{r}$.

**FIGURE 12:**
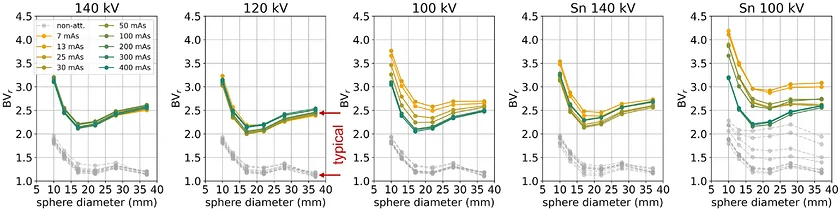
Background variability in the NEMA‐IQ spheres for different tube voltages and currents for 60 ROIs with a diameter of 37mm each. The colored curves show the background variability in a region with attenuating material surrounding the phantom with streak artefacts and photon starvation present for lower tube voltages and currents, the grey curves refer to a region without attenuating material, where no artefacts are observed.

#### 3.3.5 PET signal‐to‐noise ratio

The PET SNR followed similar trends as the SUV. The SNR ranged from 14 for the smallest NEMA‐IQ sphere with 10mm diameter and increased to 22 for the larger spheres with a diameter of 22mm, 28mm and 37mm (see Figure 13). For lower tuber voltages, for example, 100kV and Sn‐100kV, the impact of the exposure became visible, with lower currents leading to decreased SNR. The relative differences of the SNR remained similar to the difference in ${\mathrm{SUV}}_{\mathrm{mean}}$, with most protocols causing differences smaller than 1% compared to the typical clinical protocol (120kV, 50mAs). Exceptions were protocols with tube voltages of Sn‐100kV for exposures of 100mAs and below, and tube voltages of 100kV with exposures of 25mAs and below.

**FIGURE 13:**
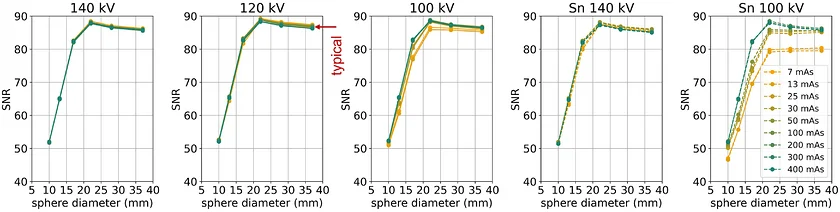
SNR for different tube voltages and currents.

#### 3.3.6 Accuracy of attenuation correction and scatter estimation

Depending on the CT image used for attenuation correction and scatter estimation, the activity registered in the ROI of the “lung” scatter insert was between 3.5% and 4.5% of the background activity, as quantified by the “lung” residual error (see Figure 14 (left)). The curves follow a similar pattern as the CT noise assessment, displaying an exponential increase in “lung” residual error with decreasing exposure. Images reconstructed with 120kV and, 13mAs and 7mAs, 100kV and exposures of 50mAs and below, and Sn‐100kV and exposures of 100mAs, which had been impacted by the photon‐starved regions in Figure 4 and Figure 9, showed larger “lung” residual errors (increase by 10%to20%), implying that the applied corrections for scatter and attenuation are less accurate if estimated from these CT protocols.

**FIGURE 14:**
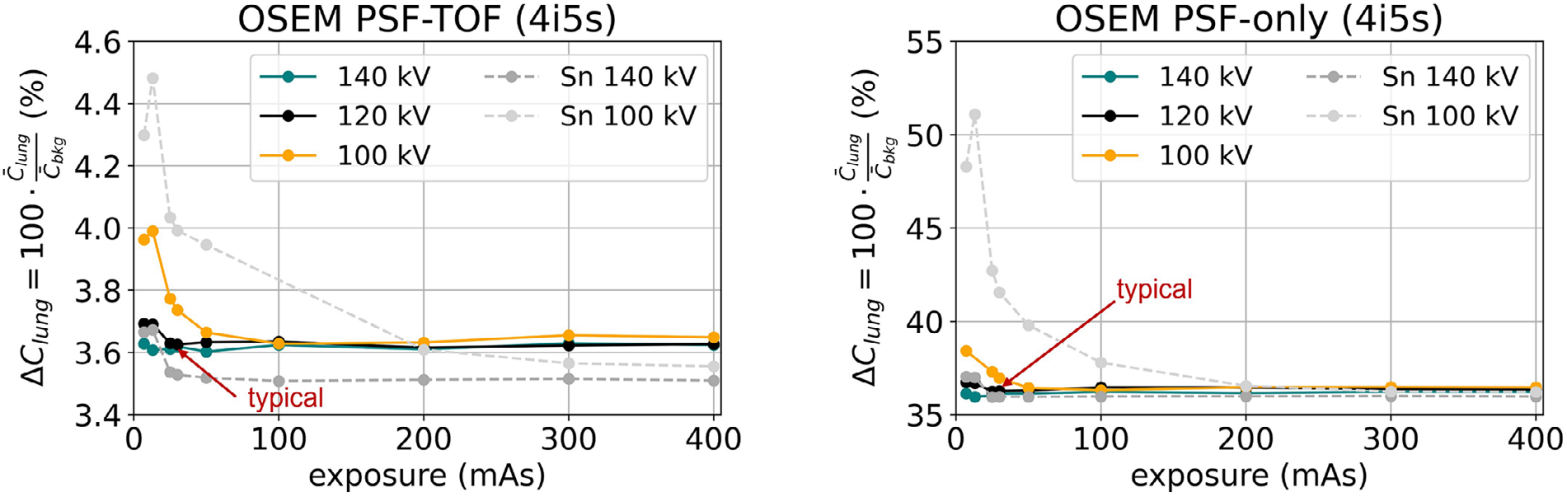
“Lung” residual error extracted from reconstructed PET images with 45 different CT protocols applied for attenuation correction and scatter estimation. (Left image) OSEM PSF‐TOF reconstruction. (Right image) OSEM PSF‐only reconstruction. Note the different scales of the y‐axis.

The “lung” residual error was larger by roughly a factor of 10 for reconstructions in non‐TOF reconstructions (see Figure 14 (right)), suggesting that TOF information and the localization of annihilation photons along the line of response are beneficial to keep the differences in the PET image due to attenuation and scatter correction at a lower level, that is the changes in “lung” residual error relative to the typical clinical protocol are reduced (see Figure 15).

**FIGURE 15:**
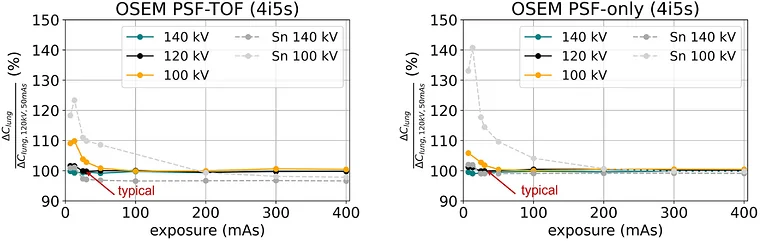
“Lung” residual error relative to the “lung” residual error of the typical clinical protocol extracted from reconstructed PET images with 45 different CT protocols applied for attenuation correction and scatter estimation. (Left image) OSEM PSF‐TOF reconstruction. (Right image) OSEM PSF‐only reconstruction.

## 4 DISCUSSION

### 4.1 Dose assessment

It is important to note that we did not use tube voltage or current modulation in this study to reduce the DLP delivered to the phantom. Firstly, the short axial length of the phantom would mainly lead to dose savings stemming from modulation of tube voltage and current because of scanning air outside of the phantom range and thus would not be representative. In addition, dose modulation can have negative effects for imaging vulnerable groups, for example when imaging the womb of a pregnant patient, where the tube current and voltage are expected to be ramped up compared to the rest of the body despite not needing high image quality in this body region in most of the cases. In contrast, for imaging adult patients, allowing tube current modulation could potentially reduce artefacts while maintaining the same overall radiation dose. The DLP savings for an adult patient are therefore potentially larger than the ones reported here using fixed tube voltages and currents. However, the absence of an air gap between the “arms” and “torso” in this phantom study might have acted favourably to reduce artefacts. If the arms are not positioned tightly to the body in an actual patient scan, but an air gap remains between torso and arms, this could potentially increase streak artefacts. Clinical studies are needed to address these effects. Another important issue to note is that the dose assessment in this study refers to the DLP only, as conversions to an actual dose with and without tin filtration can be challenging. It is possible to estimate the relative difference in radiation dose using different tube voltages with and without tin filtration.^21^, ^47^, ^48^, ^49^ Therefore, we suggest calculating the relative DLP with our typical clinical protocol as a reference point (tube voltage 120kV) and scaling the relative DLPs of protocols using different tube voltages by the reported scaling factors (see Table 2). The ED is then estimated via

(7) $${\mathrm{ED}}_{i}=k_{d}\cdot \frac{{\mathrm{DLP}}_{i}}{{\mathrm{DLP}}_{120\mathrm{kV},50\mathrm{mAs}}}\cdot {\mathrm{ED}}_{120\mathrm{kV},50\mathrm{mAs}}$$

While this might help generate an initial estimate for the actual dose savings with fixed exposures and voltages, these conversion factors must be treated with caution as they were mostly assessed for lung,^21^, ^48^ angiography^49^ and abdomen^21^, ^47^ CTs, but lack data for other body regions, for example the brain. Some groups report doses of under 0.5mSv with CT protocols using a tube voltage of Sn‐140kV and an exposure of 6mAs,^50^ where the settings closely match the protocol with a tube voltage of Sn‐140kV and an exposure of 7mAs investigated in this study.

**TABLE 2:** Empirical scaling factors to estimate the relative difference in delivered radiation dose between different tube voltages.

Tube voltage (kV)	Scaling factor $k_{d}$	References
100kV	0.87	Ha et al.^47^
120kV	1.00	Reference protocol
140kV	1.35	Lee et al.^49^
Sn‐100kV	1.1	Fong et al.^21^
Sn‐140kV	approx. 1.2	Fong et al.^21^, estimated from value for Sn‐150kV

### 4.2 CT artefacts and differences in HU

The observed streak artefacts are caused by the presence of the two marrow bones left and right of the phantom, resulting in the absorption of the low‐energy photons in the CT beam. This hardens the beam, causing non‐uniform attenuation across the phantom which displays as light and dark streaks across the image. The region starved of photons is likely to be caused by the thick layers of saline in the lower left and right of the phantom, absorbing low‐energy photons and resulting in insufficient photons reaching the CT detector especially at low exposures and voltages. Both these effects are intended in this study, given the intended resemblance of the phantom to body regions that are likely to suffer from these type of artefacts.

The observed difference in HU with changing the tube voltage is expected from the HU definition. The material attenuation coefficients are subject to changing with the photon energy. As the tube voltage will drastically change the profile of the energy spectrum, this results in different material attenuation coefficients. Whilst per definition, the HU for water will remain at 0HU, neglecting calibration drifts and reconstruction effects, the relative attenuation coefficients and hence the resulting HU will change. Regarding the use of sand to assess the quantification within bone, we are aware that many sands include silica stemming from quartz stones which are unnatural to the human body and might impact the attenuation coefficients of the material used. Since the relative HU differences for sand mixed with water follow similar differences and trends to bone, we have assumed that sand mixed with activity is suitable to assess the impact of CT protocols on the PET quantification in bone, especially if comparing the relative impact. Single acquisitions were used to extract these trends and report the HU measured for each exposure at different tube voltages in Figure 6. It is evident that the HU variation between multiple acquisition at the same voltage is smaller than the standard deviation of the HU in the selected ROI. Hence, we consider it safe to assume that single acquisitions will show reproducible trends.

### 4.3 PET quantification

For both the NEMA‐IQ spheres and the vials resembling soft tissue close to bone or a bone metastasis, the differences observed in PET quantification remained within 2% of the ${\mathrm{SUV}}_{\mathrm{mean}}$ of the typical clinical protocol (120kV, 50mAs) for most of the tested protocols. This matches findings in the literature for water‐ and bone‐equivalent material densities, which reported differences from ≤ 0.6%to6.4%.^29^, ^30^ In contrast to other reports,^29^, ^30^ we find larger differences in ${\mathrm{SUV}}_{\mathrm{mean}}$ (10%to20%) for protocols suffering from strong streak artefacts and large photon‐starved regions, when configuring low exposures and voltages (with and without tin filtration). These differences do not only occur in bone‐equivalent materials, but especially in soft‐tissue‐like materials that are positioned in‐between bones or thick layers of saline. These regimes are currently not assessed in the literature, but likely to occur in specific body regions such as hips, shoulders or the abdomen for a patient being scanned arms‐down (see Section 4.5). The observed effects and measured differences were compared to a NEMA‐IQ phantom excluding placement of the high‐attenuating bones and just placing a layer of 3cmto5cm saline on top of it. In this study, only the protocols with a tube voltage of Sn‐100kV and an exposure of 7mAs and 13mAs produced artefacts. Except for these two protocols, all measured ${\mathrm{SUV}}_{\mathrm{mean}}$ remained within 2% of the ${\mathrm{SUV}}_{\mathrm{mean}}$ of the typical clinical protocol, but this highlights the need for the proposed phantom assembly including bones and thick layers of saline or soft tissue to accurately assess the occurrence and impact of artefacts. Comparison values for the reported ${\mathrm{BV}}_{r}$, lung residual error, and SNR could not be found in the literature. It remains to be investigated whether the quantification accuracy shown here can be upheld in low‐count regimes.

### 4.4 Choice of protocol

When choosing a suitable CT protocol, three key variables need to be considered: The radiation dose delivered to the patient, the CT image quality (noise, presence of artefacts), and the PET quantification accuracy. This parameter space is mapped out in Figure 16, showing the relative difference in DLP on the *x*‐axis, the relative difference in CT noise on the *y*‐axis, and the difference in ${\mathrm{SUV}}_{\mathrm{mean}}$ color‐coded. The black and orange circles highlight protocols without and with tin filtration, respectively. It is evident that tin filtration results in lower doses at a similar CT noise level, visible by the shift of orange‐enveloped markers compared to the black‐enveloped ones along the *x*‐axis. Medium DLP and CT noise are associated with slight elevation of the ${\mathrm{SUV}}_{\mathrm{mean}}$ (less than 2%), while ultra‐low DLP protocols and high CT noise are associated with a decrease of the ${\mathrm{SUV}}_{\mathrm{mean}}$, most likely due to the presence of strong artefacts in the attenuation map. In general, it is possible to select a protocol with significant DLP decrease, marginal CT noise increase and constant ${\mathrm{SUV}}_{\mathrm{mean}}$ compared to the typical clinical protocol (120kV, 50mAs). For example, a protocol using tin filtration with a fixed tube voltage of Sn‐100kV and a fixed exposure of 200mAs would result in a substantial DLP reduction of 76.1%, while resulting in a manageable CT noise increase by a factor of 1.6 and a nonsignificant difference in PET quantification (difference in ${\mathrm{SUV}}_{\mathrm{mean}}$ < 1% for both the NEMA‐IQ spheres and the vial filled with activity and sand mimicking a bone metastasis). The choice of this example protocol excludes any protocols with heavily photon‐starved regions or strong streak artefacts, associated with larger differences in quantification, which result in much greater concern than any slight differences in the CT HU (see Section 4.3) In the end, the selection of the protocol remains subject to the clinical needs and diagnostic requirements. Recommendations based on this study are scanner‐ and phantom‐specific, however, the literature shows that similar trends are observed on standard‐axial‐field‐of‐view PET scanners with similar CT scanners.^31^ Wider generalization would require patient studies. The typical clinical protocol in this study was picked as a point of reference, as it is widely used in the field. Identifying one of the CT protocols as an objective ground truth is beyond the scope of this work.

**FIGURE 16:**
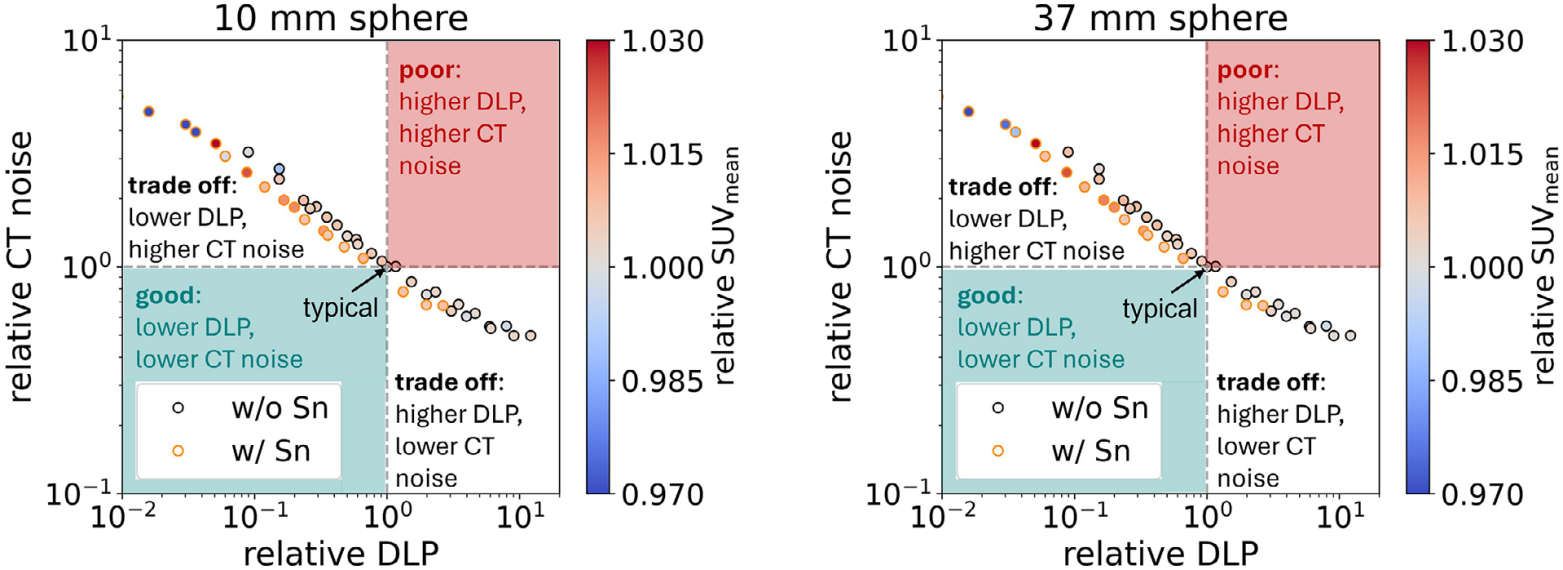
Scatter plot depicting the correlation between relative difference in DLP, relative CT image noise, and relative difference in ${\mathrm{SUV}}_{\mathrm{mean}}$ for the example of the 10‐mm and 37‐mm spheres with and without tin filtration.

It is worth noting that several groups are currently exploring alternatives to ultra low‐dose CT acquisitions, such as synthetic or pseudo‐CTs, in order to reduce the radiation burden,^51^, ^52^, ^53^, ^54^, ^55^ while the clinical availability of such prototypes is expected to be vendor‐dependent.

### 4.5 Clinical implications and limitations of this study

The resemblance of the phantom assembly to an arms‐down patient (common for imaging vulnerable groups, for example pregnant^56^ or paediatric^57^ patients) or other body regions with distinct osseous structures left and right of a larger soft tissue region, such as the hips in prostate imaging, is consistent with the observation of streak artefacts and photon‐starved regions in clinical PET/CT scans with low‐dose CT protocols. As the overall impact of these artefacts remained manageable for higher exposures, it is recommended to reduce the tube voltage (100kV) and use tin filtration (Sn‐140kV, Sn‐100kV). For protocols with tin filtration, we recommend increasing the exposure (> 100mAs) to obtain sufficient CT quality in patient studies. For protocols without tin filtration, lower exposures may still provide sufficient CT image quality without suffering from strong artefacts. These recommendations are based on the presence or absence of both photon starvation and streak artefacts in the CT image, not on achieving an as low dose as possible. Still, they result in substantial CT radiation dose reductions which is paramount to reduce the overall radiation burden for PET imaging with LAFOV PET scanners which due to their high sensitivity enable ultra‐low‐dose PET imaging for vulnerable groups and healthy volunteers. The CT radiation dose is the key parameter to be optimized to allow these groups to benefit from the advantages of LAFOV PET. It must be kept in mind that the phantom assembly is still slim compared to an adult patient (approx. 70kgto100kg), where artefacts could be even more pronounced. It is furthermore worth noting that the presence of artefacts in the image was of bigger impact on the quantification than any difference in CT HU due to a change of protocol in both soft tissue/water and bone/sand. We also recommend investigating the impact of CareDose4D and CarekV as this is expected to increase dose savings.

## 5 CONCLUSION

The recorded DLPs suggest that potential dose savings of ≥ 80%, compared to the typical clinical protocol (120kV, 50mAs), can be achieved for very low exposures. Rescaling the recorded relative DLP with scaling factors accounting for the shape of the energy spectrum with different tube voltages and with and without tin filtration might be a way to estimate ED savings for the tested protocols. The phantom assembly produced beam hardening and photon starvation in the CT image for a range of protocols. These regimes are likely to occur in specific body regions such as hips, shoulders, or the abdomen for a patient being scanned arms‐down (common for imaging vulnerable groups, for example pregnant^56^ or paediatric^57^ patients). The CT image noise decreased exponentially with the exposure and was higher in regions surrounded by attenuating materials (bone, saline). HU differences and noise were greater in bone (10%to40% difference) than in other materials (1%to30% difference). For both the NEMA‐IQ spheres and the vials resembling soft tissue close to bone or a bone metastasis, the differences observed in PET quantification remained within 2% of the ${\mathrm{SUV}}_{\mathrm{mean}}$ of the typical clinical protocol for most of the tested protocols. We found larger differences in ${\mathrm{SUV}}_{\mathrm{mean}}$ (10%to20%) for protocols suffering from strong streak artefacts and large photon‐starved regions. The same protocols showed an increased inaccuracy of attenuation correction and scatter estimation via the “lung residual error” (up to 10%to20% increase), where TOF information is beneficial to keep these changes at a low level. The ${\mathrm{BV}}_{r}$ remained within 10% of the ${\mathrm{BV}}_{r}$ of the typical clinical protocol for most tested protocols. The SNR remained within 1% of the SNR of the typical clinical protocol for most tested protocols. If diagnostic image quality is not required, CT protocols making use of tin filtration are useful to substantially reduce the CT radiation dose. For both soft tissue and bone, the presence of artefacts in the image was of bigger impact on PET quantification than any difference in CT HU.

## FUNDING STATEMENT

The PET scanner (Biograph Vision Quadra) was provided by funding from the Medical Research Council (from the UKRI Infrastructure Fund) to support the establishment of the National PET Imaging Platform (grant number MR/Y008987/1). This research was supported by the National Institute for Health and Care Research (NIHR) Clinical Research Facility at Guy's and St Thomas' NHS Foundation Trust. For open access, the author has applied a CC BY public copyright licence to any Author Accepted Manuscript version arising from this submission. The views expressed are those of the authors and not necessarily those of the NHS, the NIHR or the Department of Health.

## CONFLICTS OF INTEREST STATEMENT

Vanessa Nadig and Daniel Darian are employees of Siemens Healthcare Limited, Camberley, UK. King's College London and Guy's and St. Thomas' NHS Foundation Trust receive financial and in‐kind support from Siemens Healthineers for ongoing research collaboration projects. Alexander Hammers has received speaker's honoraries from Siemens Healthineers.

## Data Availability

Data that support the findings of this study can be made available upon reasonable request from the corresponding author.
